# The Therapeutic Potential of DNA Damage Repair Pathways and Genomic Stability in Lung Cancer

**DOI:** 10.3389/fonc.2020.01256

**Published:** 2020-07-28

**Authors:** Joshua T. Burgess, Maddison Rose, Didier Boucher, Jennifer Plowman, Christopher Molloy, Mark Fisher, Connor O'Leary, Derek J. Richard, Kenneth J. O'Byrne, Emma Bolderson

**Affiliations:** ^1^Cancer & Ageing Research Program, School of Biomedical Sciences, Institute of Health and Biomedical Innovation at the Translational Research Institute (TRI), Queensland University of Technology (QUT), Brisbane, QLD, Australia; ^2^Princess Alexandra Hospital, Brisbane, QLD, Australia

**Keywords:** lung cancer, DNA damage, DNA repair, cancer therapy, chemotherapy

## Abstract

Despite advances in our understanding of the molecular biology of the disease and improved therapeutics, lung cancer remains the most common cause of cancer-related deaths worldwide. Therefore, an unmet need remains for improved treatments, especially in advanced stage disease. Genomic instability is a universal hallmark of all cancers. Many of the most commonly prescribed chemotherapeutics, including platinum-based compounds such as cisplatin, target the characteristic genomic instability of tumors by directly damaging the DNA. Chemotherapies are designed to selectively target rapidly dividing cells, where they cause critical DNA damage and subsequent cell death (1, 2). Despite the initial efficacy of these drugs, the development of chemotherapy resistant tumors remains the primary concern for treatment of all lung cancer patients. The correct functioning of the DNA damage repair machinery is essential to ensure the maintenance of normal cycling cells. Dysregulation of these pathways promotes the accumulation of mutations which increase the potential of malignancy. Following the development of the initial malignancy, the continued disruption of the DNA repair machinery may result in the further progression of metastatic disease. Lung cancer is recognized as one of the most genomically unstable cancers (3). In this review, we present an overview of the DNA damage repair pathways and their contributions to lung cancer disease occurrence and progression. We conclude with an overview of current targeted lung cancer treatments and their evolution toward combination therapies, including chemotherapy with immunotherapies and antibody-drug conjugates and the mechanisms by which they target DNA damage repair pathways.

## DNA Damage Repair and Genomic Instability in Lung Cancer

The integrity of cellular DNA is under continual stress, receiving over 30,000 damaging events per day (4). Damaged DNA bases and DNA single-strand breaks are the most abundant types of DNA damage. Although DNA double-strand breaks are less common, they are considered as the most deleterious types of DNA damage (5). Maintaining DNA integrity is essential to prevent cancer development, which is accelerated by the accumulation of mutations. DNA breaks can arise from both endogenous and exogenous insults (6). Endogenous DNA damage can be induced by cellular processes, including somatic and meiotic recombination, and reactive oxygen species (ROS) that arise from normal cellular metabolism. Exogenous insults include cellular exposure to radiation, chemotherapeutic agents, and environmental carcinogenic compounds (7).

Lung cancers generally exhibit a unique genomic profile in contrast with other tumor types, with a high rate somatic mutation burden, second only to melanoma (8). The somatic lung cancer mutation rate was found to be much higher in smokers, 8–10 mutations/Mb, compared to <1 mutation/Mb in non-smokers, strongly supporting the causality of tobacco carcinogens (9). Highlighting the high genome instability in lung cancers, aneuploidy, (an abnormal number of chromosomes) is detected in over 60% of NSCLC cases (10) and genome duplication is observed in 30–50% of lung cancers (11).

The development of lung cancer is primarily thought to result from environmental provocation; however, there is data supporting that germline mutations in DNA repair genes increase the predisposition to the disease (12). Supporting this it has been shown that in ~2.5% of all cancer, a germline mutation in a DNA repair gene was associated with cancer development (12).

DNA repair pathways are crucial to prevent the accumulation of DNA lesions and mutations that may promote tumorigenesis through the dysregulation of cell growth and death pathways. However, the gradual loss of genomic integrity can be accelerated by environmental factors such as carcinogens from cigarette smoke promoting the development of driver mutations, including oncogene activation and/or loss of tumor suppressor function, further increasing the likelihood of tumorigenesis.

Specifically, genomic instability promotes lung cancer pathogenesis by the constitutive activation of proto-oncogenes, including the members of the EGFR (ERBB), MYC, and RAS families, along with *PIK3CA, NKX2-1*, and *ALK*. Mutations (*KRAS, EGFR*, and PIK3CA) and amplifications (*MYC, EGFR, HER2, PIK3CA*, and *NKX2-1*) commonly activate these proto-oncogenes. In addition, translocations and inversions can also occur, positioning these genes under the control of constitutively active genes such as MYC or create chimeric proteins, such as the *ALK-EML4* fusion commonly observed in lung cancers (13, 14).

Probably the most significant exogenous factor contributing to lung cancer is the exposure of lung cells to cigarette smoke, which is well-known to significantly increase an individual's risk of developing lung cancer. Cigarette smoke is recognized as a major carcinogen, identified to contain 98 individual carcinogenic compounds (15). Many of these are predicted to be mutagenic via direct interaction with the DNA (16). If cells are unable to effectively repair these lesions, mutations may arise as a result of the carcinogenic exposure, potentially promoting tumorigenesis.

Once a DNA damage event is detected, it can be repaired by one or several of the DNA repair pathways: broadly defined as base excision repair (BER); direct repair (DR); homologous recombination (HR); mismatch repair (MMR); nucleotide excision repair (NER); or non-homologous end joining (NHEJ; as shown in **Figure 2**). Defective DNA repair mechanisms in cancer cells are often associated with poor patient prognosis due to enhanced disease progression. However, defects in DNA repair machinery may also provide an avenue to specifically target cancer cells due to increased sensitivity to anti-cancer therapies (17).

## DNA Repair Pathways

### Nucleotide Excision Repair

**Figure 1:**
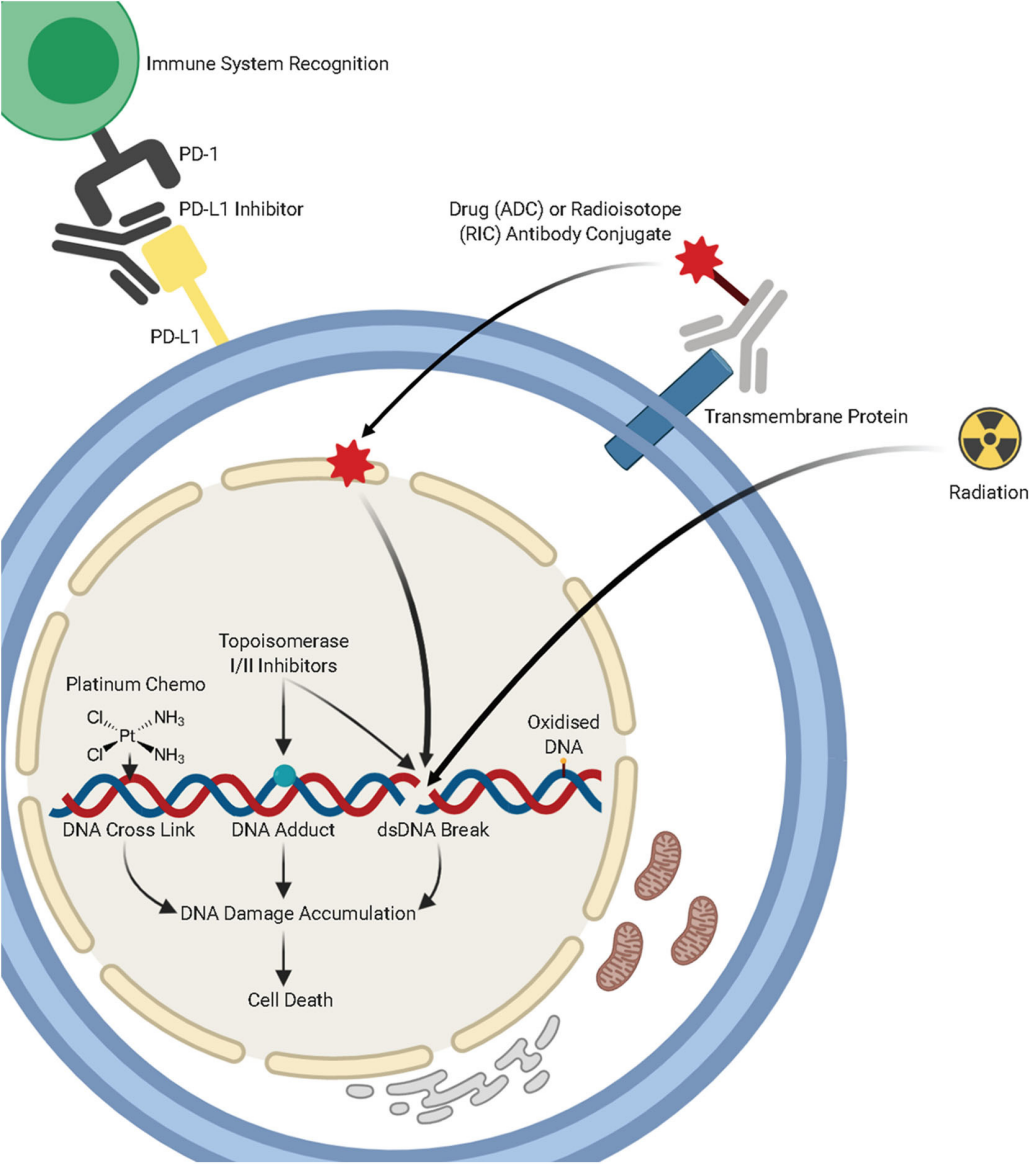
A summary of lung cancer treatments, including chemotherapy, radiotherapy, immunotherapies, and antibody-drug conjugates and the mechanisms by which they target DNA damage repair pathways. Figure created with Biorender.

Bulky DNA adducts caused by UV light and chemotherapeutic agents, such as Cisplatin, are repaired primarily through the nucleotide excision repair pathway (NER). This pathway is composed of a series of enzymatic reactions which are facilitated by over 30 proteins. Cisplatin and other platinum compounds bind to the DNA and form adducts, which lead to intrastrand or interstrand crosslinks (Figure 1A). These bulky adducts cause distortion of the DNA helix, blocking the replicative DNA polymerases and subsequently DNA replication and require the NER pathway (or the MMR pathway) for repair. These chemotherapeutic agents exploit differences in cellular proliferation and DNA repair pathways in cancerous cells to specifically target tumors.

As with the other DNA repair pathways, NER is a stepwise process initiated by DNA damage recognition; followed by recruitment of the pre-incision protein complex and DNA unwinding. This allows for excision of the damaged fragment and subsequent DNA repair and ligation [reviewed in (7)].

Depending on the DNA damage recognition step, the NER pathway is divided into two sub-pathways. Transcription coupled NER (TCR) is initiated by the stalling of transcription by RNA polymerase II promoting the subsequent recruitment of the Cockayne syndrome (CS) complementation group A and B to initiate repair (18, 19). During global genome NER (GGR), the XPC/hHR23B, DDB1, and DDB2/XPE complexes recognize the DNA lesions. Following damage recognition, TCR and GGR follow the same mechanism. The DNA helix is unwound by the TFIIH complex to enable access to the pre-incision complex (XPD, XPB, XPA, and XPG) (20, 21), opening the DNA double strand for the recruitment of RPA The damaged DNA is then excised, leading to removal of a patch of 24–32 base pairs, facilitated by XPG and the CPF-ERCC1 (excision repair cross-complementing enzyme group 1) endonucleases. DNA synthesis replaces the excised DNA in conjunction with PCNA and RPC, followed by the repair of the backbone through DNA ligase I (18).

Single nucleotide polymorphisms (SNPs) in NER associated proteins have been shown to have clear links to lung cancer patient survival and responsiveness to treatments. SNPs in NER proteins including ERCC1, ERCC6, POLD2, POLE, and XPA are associated with progression free survival. SNPs in other NER proteins, including ERCC6, GTF2H4, GTF2HA, MAT1, POLD1 are associated with overall survival (22, 23). Furthermore, decreased expression of XPG/ERCC5 and CSB/ERCC6 has been demonstrated to increase the risk of lung cancer (24).

Cisplatin sensitivity has been associated with SNPs and gene expression within the NER pathway. ERCC1 (excision repair cross-complementing enzyme group 1) is one such protein and functionally associates with XPF (xeroderma pigmentosum complementation group F) to incise damaged DNA (25).

ERCC2, another NER repair protein, is a helicase that unwinds DNA strands in the vicinity of a damaged site. ERCC2 mRNA expression has been shown to significantly correlate with cisplatin resistance in preclinical studies (26, 27). Cumulative evidence indicates that polymorphisms of the NER repair gene, ERCC5, could serve as pharmacogenomics biomarkers (28). In addition, a polymorphism in RRM1, a large regulatory subunit of ribonucleotide reductase, involved in the NER pathway, has been identified as a promising prognostic biomarker (29).

### Mismatch Repair

DNA mismatch repair (MMR) is a conserved process responsible for the recognition and repair of mispaired bases generated during DNA replication and other DNA damage repair pathways. In addition to base mismatch, MMR can recognize other DNA lesions including DNA crosslinks induced by chemotherapeutic agents (30, 31).

**Figure 2:**
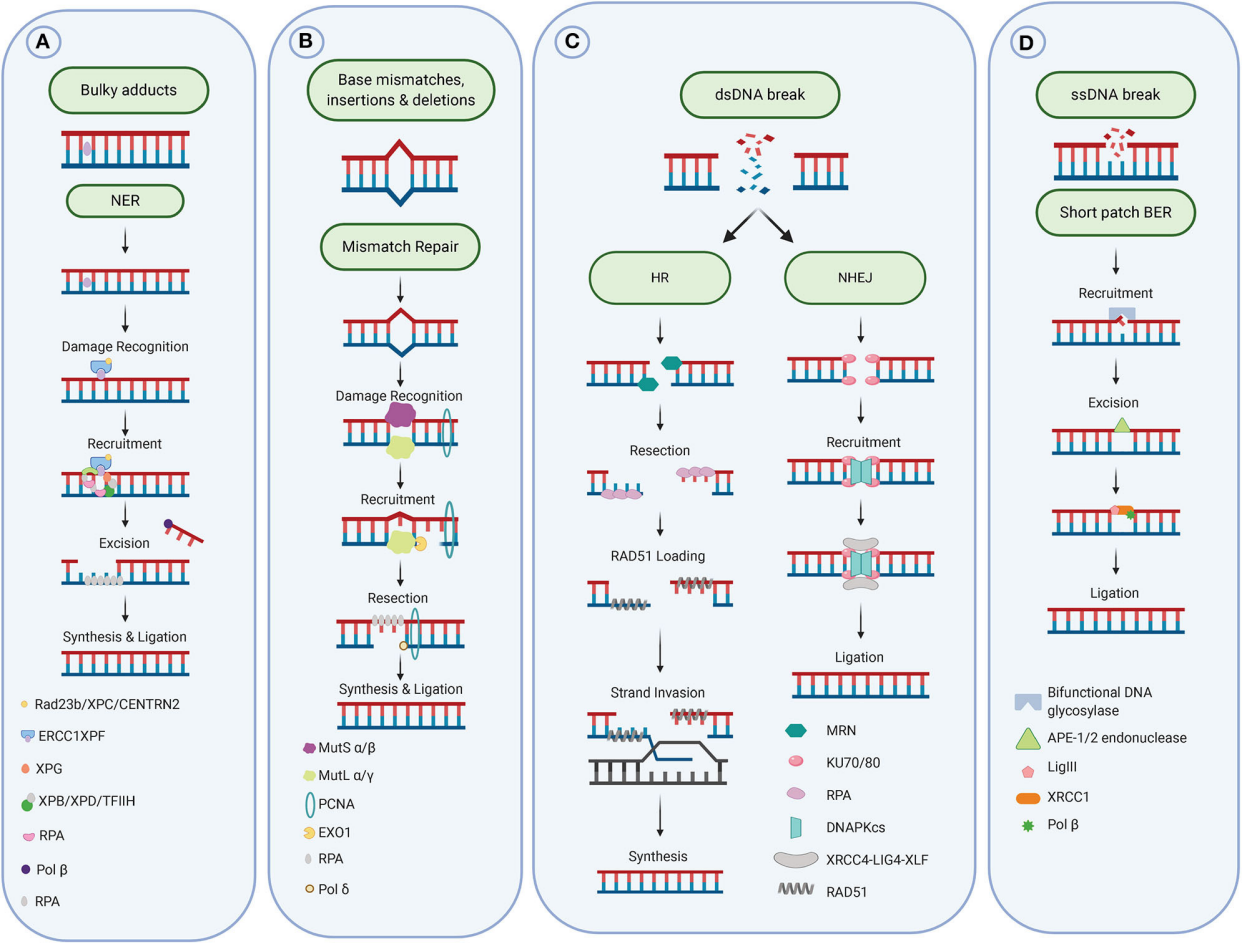
DNA repair pathways. **(A)** Repair of bulky adducts via the Nucleotide excision repair pathway. **(B)** Repair of mismatches via the mismatch repair pathway. **(C)** Repair of DNA double-strand breaks via the homologous recombination and non-homologous end-joining pathways. **(D)** Repair of DNA single strand breaks and damaged and oxidized bases via the base excision repair pathway. Figure created with Biorender.

The MMR process consists first of the damage recognition by the sliding clamp MutSα (MSH2/MSH6 heterodimer, for base mismatch) or MutSβ (MSH2/MSH3 heterodimer, for base insertion/deletion), followed recruitment of the MutLα complex (formed by MLH1 and PMS2) for the incision step. Exonuclease 1 (EXO1) is then recruited to excise the damage, leaving a gap filled by DNA polymerase δ (Polδ). Finally, DNA ligase I closes the remaining nick on the newly synthesized strand [see for review (32), Figure 2B].

Inactivation of MMR can have wide ranging consequences due to its involvement in the repair of base substitution mismatches and insertion–deletion mismatches that escape DNA polymerase proofreading during replication (33). MMR proteins function in the activation of cell cycle checkpoints, supressing DNA lesions, and the initiation of apoptosis (34).

MMR proteins can be inactivated without causing cell lethality; however, this can lead to a significantly increased rate of genome-wide point mutations, resulting from unrepaired DNA synthesis errors. The mutator phenotype conferred by this loss of MMR activity contributes to the initiation and promotion of multi-stage carcinogenesis (35). MMR is dysregulated in non-small cell lung cancer, most frequently from mutations in the MSH2 and MLH1 genes, which are responsible for recognition of the mispaired nucleotides, deletions/insertions, and cisplatin-induced interstrand cross-links (36, 37). Decreased expression of MSH2 has been associated with increased cisplatin sensitivity (38).

In addition to mutations, mismatch repair may also be downregulated through epigenetic changes including hypermethylation of the MLH1 promoter, a defect occurring in 69% of non-small cell lung cancers, although it should be noted that this has also been disputed (39, 40). The prognostic significance of expression and methylation changes in these genes in non-small cell lung cancers is not well-understood and requires further study (41).

### Base Excision Repair and Single-Strand Break Repair

Oxidative DNA damage has been shown to be a driver of carcinogenesis (42). Damage to bases within DNA requires the base excision repair (BER) pathway for the effective repair of these lesions (Figure 2D). The BER pathway removes small covalent modifications such as those generated from reactive oxygen species (ROS) as a result of cellular metabolism or endogenous damage (43). Guanine, due to its chemical composition, is the most frequently oxidized base and following oxidation forms 8-oxo-7, 8-dihydro-guanine (8-oxoG) (44, 45). These lesions may result in G:C to A:T transversion during replication, due to the mis-pairing of 8-oxoG with cytosine of adenine nucleotides. Significantly these transversions are suggested to be one of the most common mutagenic features observed in many cancers (46, 47). Other types of damage involve alkylation or deamination (48).

The repair of these lesions involves the recognition and removal of the adduct by a specific DNA glycosylase (mono- or bi-functional), such as OGG1, NTH1, UNG, SMUG1, TDG, NEIL1, NEIL2, or NEIL3 [see for review (49)]. An AP-endonuclease, most commonly APE1, then catalyzes the hydrolysis of the site to generate a DNA single-strand break. This then activates the single-strand break repair pathway, which stimulates the poly-ADP ribose activity of PARP1. PARP1 uses NAD+ to catalyze the addition of long, branched PAR chains to onto serine, tyrosine and glutamic acid residues in the PARP1 automodification domain, in a process known as auto-ADP-ribosylation (50–54). This leads to further activation of PARP1 and thereby stimulates the PARP1-mediated poly-ADP-ribosylation of other repair proteins such as XRCC1 (X-ray repair cross-complementing protein 1). The nucleotide is then replaced by a DNA polymerase β and finally DNA ligase III repairs the DNA backbone (55). This process replaces a single nucleotide; however, long-patch BER is also possible where DNA polymerase δ/ε with PCNA replace a short sequence containing the damage (~5 nucleotide). The endonuclease FEN1 resects the generated flap before final nick ligation by DNA ligase I. The reconstitution of long-patch-BER has demonstrated an absolute requirement for the endonuclease activity of FEN1, which, is often upregulated in cancers (56).

The reliance of tumor cells on BER pathways makes an attractive target for cancer therapy. Supporting this, elimination of N-Methylpurine DNA glycosylase (MPG) or inhibition of APE1 has been shown to increase sensitivity of cancer cells to alkylating chemotherapeutics (57). Several APE1 and Pol β inhibitors have also been developed and proved to be effective in cell line and mouse models; however, these have not yet progressed to human trials (58). PARP1 inhibitors have been approved as a human cancer therapy and will be discussed further in this review.

Mutations in key BER genes have been associated with an increased risk of lung cancer and decreased patient survival (59). Variants in the BER glycolyase and AP-endonuclease OGG1 and APEX1, respectively, have been shown to be associated with an increased risk of lung cancer (60–62). Variants in BER proteins have also been shown to associate with decreased patient survival, including; MDB4, a DNA binding protein, APE1, the primary endonuclease, OGG1, XRCC1 and Polymerase β variants (60–64).

### DNA Double-Strand Break Repair

Regarded as potentially the most severe form of DNA damage, DNA double-strand breaks (DSBs) are induced by a variety of mechanisms. These include exogenous factors such as ionizing radiation and chemotherapeutic agents, or endogenous sources such as faulty DNA replication and oxidative stress induced by reactive oxygen species (ROS) during normal cellular metabolism (65). It is predicted that over 10 DSBs are induced per cell, per day and this can have severe consequences for cells, as failure to repair dsDNA breaks can lead to senescence or apoptosis. Furthermore, incorrect repair can result in genomic instability or mutation of critical regulatory genes and subsequently, tumorigenesis (66). Non-small cell lung cancers frequently exhibit mutations or loss of essential components of the DSB repair pathways (67, 68). Two main pathways are involved in the repair of DSBs; Homologous Recombination (HR) and Non-Homologous End Joining (NHEJ) [Figure 1C, (69)].

The two pathways are generally distinct from each other, however some of the repair machinery is involved in both pathways. NHEJ does not require a homologous template and involves the ligation of the two free DNA ends, without extensive resection, in contrast to HR. This ligation is initiated by the tight binding of the ring-shaped Ku70/Ku80 heterodimer to the two DSB ends to promote recruitment of DNA-dependent protein kinase (DNA-PK) and form an active catalytic complex (69). The bridging of the dsDNA break by the complex may initially enable DNA digestion or gap-filling by recruitment of the proteins Artemis (70), XLF (71), and PAXX (72), followed by induction of the Ligase IV/XRCC4 complex's DNA end ligation activity (66). The configuration of the DSB ends can lead to alternative NHEJ pathways [see for review (73)]. This process, which functions in all stages of the cell cycle, can result in the joining of cut DNA fragments from either a single gene or entirely separate chromosomes. Therefore, NHEJ is generally regarded as a more error prone method of DSB repair. NHEJ has been implicated in DNA translocation from one region or chromosome to another, having the potential to result in uncontrolled cell growth.

In late S and G2 phase, mammalian cells can repair dsDNA breaks by HR due to the availability of a homologous sister chromatid in close proximity to use as a template. HR requires the high fidelity matching of individual sequence bases to allow more accurate DNA repair than NHEJ, and without loss of bases (1, 74, 75). HR is initiated by the ATM-dependent recruitment of the Mre11-Rad50-NBS (MRN) complex. This serves to further activate ATM and promotes the further recruitment of other repair proteins, including MDC1, to the site of the break (76, 77). The MRN complex then resects DNA up to 3 kb from the dsDNA break site and in conjunction with Exo1 and Dna2, digests the DNA strand between the nick and DSB (78–80), exposing a section of single-stranded DNA (ssDNA) to which replication protein A (RPA) rapidly binds, promoting the recruitment of BRCA1 (81). Under the control of the BRCA1 and BRCA2 proteins (82), Rad51 then facilitates the search for a homologous DNA sequence and promotes the strand invasion to generate a D-loop. The resynthesis of the damaged strand is then completed by DNA polymerase eta, using the homologous sister chromatid as template (83). The resulting Holliday junction is then resolved, by the 3′-flap endonuclease MUS81-EME1 or Gen1 resolvase (84, 85). Extensive degradation has also been observed at some DSBs, resulting from other mechanisms of repair called alternative-non-homologous end joining and single-strand annealing, both of which have the potential to cause mutagenic deletions (86, 87). However, it remains to be determined whether these alternative mechanisms of repair contribute toward lung tumorigenesis.

Similarly to many other cancer types, lung cancers display a high level of mutations in the tumor suppressor gene, TP53. TP53 has multiple faucets to its role in the maintenance of genome stability, including responding directly to DNA damage to promote repair and cell cycle arrest, the transcriptional regulation of DNA repair genes and the induction of apoptosis. In the absence of TP53 cells accumulate DNA damage and resist cell death. TP53 has been shown to be mutated in around 50% of all non-small-cell lung cancers (NSCLCs) and over 90% of small cell lung cancers (SCLCs). Since the presence of TP53 mutations have been detected in preneoplastic lesions in the lung, it has been hypothesized that the mutation of TP53 is likely to be an early event in the development of lung cancer (88).

In addition to TP53 mutations, mutations in the DSB repair kinase ATM, were identified in 6.12% of NSCLC. Mutations were also prevalent in other key DNA repair proteins including, TP53, BRCA2, EGFR, and PARK2 (12). There was an increased incidence of familial cancer syndromes associated with these mutations (89). Germline mutations in DNA repair pathway genes, similar to other solid tumors, are the most common subclass of genes associated with an increase in NSCLC predisposition (12).

Mutations in several DNA damage response genes, including PARP1, BRCA1, ATM, and TP53 have been shown to be associated with cancer progression and metastasis. The presence of DDR gene mutations has been linked with an increased tumor mutational burden in NSCLC. This study also showed that mutations in DNA repair genes were not mutually exclusive as 77% had a mutation in two or more genes associated with DNA repair. PARP1 and ATM mutations increase metastatic potential, likely due to their association with SNAIL-1, a master regulator of the epithelial-mesenchymal transition required for metastasis. Mutations in BRCA1 disrupt DSB repair via HR, increasing the mutation rate, and subsequently increasing the risk of tumorigenesis (90–92).

Mutations in DNA repair genes have been associated with a differential tumor response to cancer therapy. Mutations or changes in expression of genes that have been associated with chemotherapy sensitivity of NSCLC include TP73, MDM2, PTWN, PIK3, DNPK1, and DNA-PKcs (93). The treatment of NSCLC tumors possessing DNA-PK, TP53, and PTEN mutations with radiation similarly alters sensitivity in a mutant dependent manner (93). Mutations of MDM2 and TP53 have been associated with an increase in patient survival in lung adenocarcinoma (93).

Lung cancer has been well-characterized as possessing one of the most aggressive mutation rates of all cancers. Supporting this an average mutation rate of 4.21 mutations per megabase was identified in a screen of somatic mutations in protein kinase genes from 210 cancer cell lines (46). Sequencing of 188 primary lung adenocarcinomas identified 26 mutated genes, including several known tumor suppressor genes including TP53, KRAS, CDKN2A, and STK11 (LKB protein). High mutation rates in DNA repair genes, including CDNK2 (p16) and RB were also shown via whole-genome and transcriptome sequencing of lung cancers (9, 40). As mentioned above mutations in STK11/LKB1 and consequential disruption of AMPK signaling pathways are amongst the most frequent aberrations in lung cancers [reviewed in (94)]. Both LKB1 and AMPK have been suggested to have roles in DNA repair pathways, however the full extent of the influence of LKB1 downregulation on DNA repair pathways in lung cancer has not yet been explored (95, 96).

Translocations, which can result from errors in repairing DSBs, are also common in lung cancers, with gene fusions in the tyrosine kinases ALK and ROS1 being the first identified, targetable driver rearrangements in NSCLC. Fusions in other kinases have also been established as targetable, oncogenic drivers, including RET, NTRK, EGFR, and BRAF (97).

## DNA Repair Pathways as Therapeutic Targets

The recommended frontline treatment for patients with stage I–III NSCLC is surgery. For inoperable locally advanced tumors, the current standard of care involves concurrent radiotherapy and doublet chemotherapy followed by 1 year of adjuvant immunotherapy, Durvalumab. Therapies for the treatment of advanced lung cancer have become more targeted to the individual tumor, utilizing advances in molecular target technologies based on genomic abnormalities detected in the tumor tissue. It is estimated that up to 69% of patients with advanced NSCLC could have alterations in one or more molecular targets that could guide their treatment (98). These include EGFR activating mutations, KRAS, BRAF, HER2, and MET mutations, ROS1, ALK, RET, and NTRK rearrangements. EGFR, ALK, ROS-1, and BRAF positive tumors now have clinically applicable targeted specific therapies [reviewed in (99)] which offers superior patient outcomes and an improved toxicity profile compared to standard platinum doublet based chemotherapy. Clinical trials are currently underway investigating compounds that specifically target KRAS, HER2, MET, RET, and NTRX alterations in NSCLC. These compounds, if proven efficacious, will dramatically expand the treatment landscape for these patients. Single agent pembrolizumab, platinum-based doublet therapy with or without immunotherapy are current options in the first line for patients that do not fit an identified targeted therapy. Treatment decisions on the most appropriate choice will have to be balanced across a number of factors including assessment on the extent of tumor PD-L1 expression, patient co-morbidities, patient performance status, extent of disease as well as patient preference. This, together with other agents targeting DNA repair and/or genome instability, along with the emerging immunotherapies are discussed further, below.

## Chemotherapies—DNA Damaging Agents

### Platinum Therapy

Platinum doublet therapy has been a frontline therapy for lung cancer since the 1990's. This therapy consists of platinum compounds in combination with several third generation chemotherapeutic drugs, including; cisplatin/carboplatin in combination with gemcitabine, pemetrexed, docetaxel, or paclitaxel (100). It has been generally accepted that the main mechanism of action of platinum compounds as a cancer therapy is by crosslinking the purine bases within DNA, causing DNA damage. In rapidly growing cancer cells this leads to inhibition of DNA replication, cell division, and eventual cell death. Other suggested contributions of platinum compounds to cellular toxicity also include; oxidative stress, modulation of calcium signaling, and activation of several other kinases and signaling pathways [reviewed in (101)]. The emergence of personalized therapies and the toxicity and subsequent side-effects of platinum therapy have led to a reduction in their use. However, until a more effective treatment is found for tumors without an identified biomarker mutation or translocation, platinum treatment is likely to remain a mainstay of lung cancer treatment.

### Topoisomerase Inhibitors

Topoisomerase inhibitors are commonly used in combination with platinum agents for treatment of small cell lung cancer (SCLC). First line treatment for SCLC widely involves the topoisomerase II inhibitor, etoposide in combination with platinum therapy. The topoisomerase II enzyme functions to cleave double-stranded DNA and topoisomerase II inhibitors such as etoposide inhibit this activity leading to stable protein-linked DSBs in DNA and subsequent cell death in rapidly dividing cancer cells (102). Topoisomerase I functions to induce single-strand breaks in the DNA to reduce torsional stain on the DNA helix. Relapsed refractory SCLC is commonly treated with the topoisomerase I inhibitors topotecan or irinotecan, following resistance to platinum and topoisomerase II targeting therapies. Inhibition of topoisomerase I, by topotecan or irinotecan prevents repair of these single-strand breaks that are then converted into double-strand breaks in the S-phase of the cell cycle leading to tumor cell death.

### DNA-Damage Targeted Therapies

Despite the advances made in “personalized medicine” for the treatment of lung cancers, new treatments have generally not been suitable for the majority of patients, due to a lack of mutations identified in targetable genes. Of further concern, patients inevitably develop resistance to these targeted therapies through an additional mutation in the target gene or initiation of a downstream signaling pathway that stimulates tumor growth. Therefore, although significant progress has been made in lung cancer treatment in recent years, new treatment strategies are urgently required. Several proteins involved in the DNA damage response have been identified as emerging targets for cancer treatment, including; PARP1, ATR, and Chk1.

### PARP Inhibitors

The term “synthetic lethality” describes how perturbation of one gene is compatible with cell viability; however, simultaneous disruption of two genes results in cell death (103). In cancer treatments, synthetic lethality can be used to exploit tumor-driven mutations and protein expression alterations to induce cancer-specific cell death [reviewed in (104)]. This treatment specifically targets the tumor cells over the normal cells, which reduces toxic side-effects for patients and improves quality of life during treatment.

The most recognized example of synthetic lethality used in cancer therapy is the use of PARP inhibitors in homologous recombination deficient tumors (105, 106). Inhibition of the PARP enzymes results in PARP immobilization at DNA single-strand breaks and homologous recombination repair is required for replication forks to bypass this lesion. Diminished capacity to complete functional homologous recombination confers cell death following treatment with PARP inhibitors. The breast cancer associated proteins BRCA1/2 function in the repair of DNA via homologous recombination; therefore, PARP inhibitors are approved for treatment of BRCA1/2 mutated breast, ovarian, and pancreas cancer (107). The first PARP inhibitor to be approved by the European Medicines Agency in 2014 was Olaparib (Lynparza; AstraZeneca, London, UK), which is currently used as maintenance therapy for patients with BRCA1/2 mutated ovarian cancer following platinum-based chemotherapy. Subsequently, Niraparib and Talazoparib were also approved for use in a maintenance therapy setting. Several other potent PARP inhibitors are in late clinical trial development including, Veliparib. There are 416 PARP inhibitor clinical trials currently listed on clinicaltrials.gov, including 40 in lung cancer, indicating the potential of this treatment.

Although PARP inhibitor treatment in patients with a range of tumor types with germline BRCA1/2 mutations have shown to be effective, such mutations are only found in ~5% of patients with lung cancer (108). It is now clear that clinical efficacy would be beneficial beyond this niche population of patients. The need for a definitive biomarker for PARP inhibitor sensitivity has been well-documented (109) and a number of strategies have been explored to predict tumor sensitivity to PARP inhibitors, including unsuccessful attempts to identify predictive biomarkers for homologous recombination-deficient tumors (110). In terms of lung cancer, it has been suggested that high levels of DNA repair inhibiting proteins, such as SLFN11, and low levels of DNA repair promoting proteins, including ATM, may be a superior predictive biomarkers to BRCA1/2 mutations in small cell lung cancer (111, 112).

Significantly, tumors with mutations in the DNA repair protein PTEN account for 4–8% of all NSCLCs and it has been shown that PTEN mutant tumor cells are sensitive to PARP inhibitors, expanding the number of lung cancer patients this therapy may benefit (113, 114). While PARP inhibitors use in BRCA1/2 mutated tumors remains the best characterized treatment based on synthetically lethality, numerous other synthetic lethal interactions have been identified using RNAi screens, based on other mutations found in lung cancer, including mKRAS and EGFR mutations [summarized in (115)].

Although several combination treatment lung cancer clinical trials, with PARP inhibitors are underway, these trials are still in their early stages and the majority data or trial outcomes are not publically available. However, a recent trial published data, showing the safety and activity of a combination treatment of the PARP inhibitor Olaparib with the DNA damaging alkylating agent temozolomide in patients with relapsed SCLC. The combination was confirmed as safe and active, with an overall response (defined as >30% decrease in the sum of the longest diameter of target legions) in 41.7% of patients and median overall survival of 8.5 months (116). It has also been suggested that a combination of PARP1 inhibitors with immunotherapies may also be an effective combination treatment for lung cancers, particularly in tumors with other DNA repair defects, such as ERCC1 deficiency (117).

PARP inhibitors have also been implicated as a radiosensitizer in NSCLC. Since radiation therapy is the first-line treatment in patients with locally advanced NSCLC, this could have significant implications for their treatment (118, 119).

## The DNA Damage Response and Immunotherapies

### Immune-Checkpoint Inhibitors

The treatment of lung cancer through immunotherapies, including immune checkpoint inhibitors, and targeted antibodies, has dramatically expanded over recent years. Checkpoint inhibitors which target PD-1 and PDL-1 are considered a standard first and second-line treatment in lung cancer. These immunotherapies block the PD-1 checkpoint to enable the immune system to recognize and target cancer cells. There are now multiple antibodies that are raised against anti-PD1 or anti-PD-L1, including Atezolizumab, Avelumab, Durvalumab, Pembrolizumab, and Nivolumab (120).

Although immunotherapies do not directly target DNA damage repair, DNA damage, and genome instability has been shown to induce changes in the tumor microenvironment and stimulate the generation of neoantigens on cancer cells, increasing the tumor response to immunotherapies. This has led to the hypothesis that DNA damaging agents, such as cisplatin, may increase the efficacy of immunotherapies. Indeed, clinical and preclinical data have shown that chemotherapy can induce PD-L1 expression on tumor cells (121–123).

Testing of PD-L1 expression has rapidly become standard for newly diagnosed patients with advanced NSCLC. A high number of mutations in a tumor, known as the tumor mutation burden, is also associated with an increased response rate to immunotherapy. Similarly, microsatellite instability, also associated with genome instability, is also linked with a better response to immunotherapy. Many commercial laboratories now offer a comprehensive gene sequencing report comprising of the PD-L1 expression, tumor mutation burden, and microsatellite instability status of tumors (124).

In light of the above, the treatment of lung cancer patients with immunotherapies in combination with chemotherapy has shown improvement in patient survival and tumor response rate as such it has now become the standard of care for first-line treatment in several lung cancer subtypes (125–128). Pembrolizumab as a single agent was compared to standard platinum doublet chemotherapy in the first line setting in patient with advanced NSCLC in those with a tumor PD-L1 expression >50%. Updated analysis revealed a median overall survival improvement of 15.8 months in favor of pembrolizumab reported in this group. Based on this study single agent pembrolizumab is favored over platinum based chemotherapy in those with a PD-L1 >50%. A phase 2 randomized study using the combination of pembrolizumab plus carboplatin and pemetrexed demonstrated an objective response rate of 55% in the combination compared to 29% in chemotherapy alone, p:0.0016, supporting the use of a DNA damaging agent in combination with immunotherapy (129). A later phase III randomized placebo controlled trial confirmed the addition of pembrolizumab to chemotherapy led to a significant improvement in median progression free survival (8.8 vs. 4.9 months) and overall survival (12 months overall survival: 69.2 vs. 49.4%) in advanced non-squamous NSCLC. Similar findings have also been observed in another double-blind phase III trial in treatment-naive patients with metastatic squamous NSCLC. In this study, pembrolizumab was combined with carboplatin and either paclitaxel or nab-paclitaxel. In the total population median overall survival was 15.9 months in the combination (chemo-immunotherapy) vs. 11.3 months in the standard arm (130). This constitutes level one evidence for the use of combination chemo-immunotherapy and is considered as an option for the first-line treatment of patients in squamous and non-squamous advanced NSCLC, irrespective of the tumor PD-L1 status (131).

### Antibody Drug Conjugates

Antibody-drug conjugates (ADCs) are a rapidly developing area of targeted therapy in lung cancers [reviewed in (132)]. ADCs consist of monoclonal antibodies that are covalently bound to a cytotoxic chemical. These immuno-conjugates are designed to have greater cancer cells toxicity whilst minimizing off target effects on normal cells. The ADCs are now in their third generation through linker optimization, which allows for lower de-conjugation rate in circulation whilst still having a potent impact on cancer cells.

The development of ADCs is limited by the identification of a specific target antigen on the cell surface which has high expression in tumors and low or no expression in normal tissue. The toxic warhead is still a limiting factor based on a small number of cytotoxic drug families, similarly to other cancer therapies, inducing DNA damage or inhibiting microtubule formation. Difficulties arise with the selection of drugs with requirements for retaining potency following linkage whilst maintaining solubility. Compounds that target DNA include Calicheamicins which induce DNA double-strand breaks, Duocarmycins which alkylate DNA, Benzodiazepines which bind DNA to induce crosslinks and Camptothecin analogs inhibiting DNA topoisomerase I (119, 133, 134). Other ADC warheads include Auristatins, Maytansinoids, and Tubulysins which aim to prevent cancer cell proliferation by inhibiting tubulin assembly (135–138).

Several ADC have been developed that contain compounds that induce DNA damage, as discussed below. These ADC have shown promise for potential treatment of lung cancer, however it should be noted that none of these are currently licensed for use. For example, Sacituzumab Govitecan (IMMU-132) which utilizes an antibody against the transmembrane glycoprotein, Trop-2 (which is highly expressed in epithelial malignancies), conjugated with SN-38, the active metabolite of the topisomerase I inhibitor, irinotecan. In preclinical models, IMMU-132 was shown to deliver 136 fold more SN-38 than irinotecan (139). Clinical trials have shown an overall response rate of between 14 and 31% with IMMU-132 in NSCLC and SCLC (139).

SGN-15 is an ADC targeting topoisomerase II, using the drug Doxorubicin linked to a monoclonal antibody against the carbohydrate antigen Lewis-Y. In a phase II trial comparing SGN-15 in combination with docetaxel to docetaxol treatment alone in NSCLC patients, the overall response rate was 6% for the combination vs. 21% for docetaxol alone. However, the overall survival was greater in the combination treatment (140).

Rovalpituzumab Tesirine (Rova-T) consists of an antibody against delta-like ligand DLL-3 conjugated to pyrrolo-benzodiazepine dimer toxin (a DNA damaging agent). In a phase I trial, SCLC patients had a 18% overall response rate to Rova-T. An improved response rate of 38% was also observed in patients with high DLL3 expression (141). Another phase II trial showed a 14% overall response rate in SCLC patients with DLL3 high expression and 12% overall (142).

Although DNA damage inducing ADCs are not currently approved for treatment of lung cancer, the combination of ADCs with other therapies is under investigation. The treatment of patients with immunotherapy through the targeting of programmed cell death 1 ligand 1 (PDL1) or cytotoxic T lymphocyte antigen 4 (CTLA4) along with ADCs, has shown potential in several tumor types. ADC targeted therapy is rapidly advancing but there is still much to learn in terms of resistance and toxicity before this class of therapy can be fully utilized.

### Radiotherapy

Radiation therapy is an effective anti-cancer therapy which induces DNA damage by targeting the DNA to induce multiple forms of damage, including double and single-strand breaks and oxidative lesions. Furthermore, indirect ionization involves interaction with water molecules surrounding DNA to produce radical oxygen species (ROS). These ROS also generate further DNA single-strand breaks, DNA DSBs, and oxidized DNA bases, this leads to cell death in genomically unstable tumors (143).

Radiation therapy is commonly utilized in the treatment of several cancers, including lung cancer. It is still considered a first-line therapy in the treatment of non-small cell lung cancer (NSCLC). However, small cell lung cancer is primarily treated with chemotherapy, although combination radiotherapy and chemotherapy is used as a second line therapy (144). Radiation therapy for lung cancer has evolved over time to increase the accuracy of the X-ray dose delivered to the tumor, subsequently decreasing toxicity to adjacent tissues (145).

Dose fractionation and 3-Dimensional conformal radiation therapy (3DCRT, using CT images) significantly improved radiation treatment (146). The development of multileaf collimators allowing modulation of the X-ray beam dose-rate led to intensity-modulated radiation therapy (IMRT, characterized by a static delivery) and volumetric-modulated arc therapy (VMAT, with a dynamic delivery), which improved the target volume accuracy and led to less organ toxicity (147, 148).

Potential mechanisms of radiation resistance include mutations in EGFR and RAS, increased expression of MDM2 and Livin α, or decreased TP54I3 expression (149, 150). Various XRCC1 mutations have been identified to increase and decrease radio sensitivity in NSCLC (43, 151, 152). Blood-based microRNAs (miRNAs) have been identified as potential biomarkers to elucidate the tumor response to radiotherapy (153).

The field of radiation therapy is still evolving, notably with the development of stereotactic ablative radiation therapy [SBRT or SABR, used by the CyberKnife system (154)], where a very high dose is locally delivered using 3DCRT or IMRT (155). Radiation therapy can also be used in combination with sensitizing drugs (156). The molecules used target DNA [such as Cisplatin (157) or Paclitaxel (158)], DNA repair (PARP inhibitor) (159), or growth factors receptors (e.g., Epidermal Growth Factor Receptor blockade by the drug Cetuximab) (160).

### Radioimmunoconjugates

Similarly to some ADCs, radioimmunoconjugates (RICs) are designed to induce DNA-damage specifically in tumor cells, in order to induce cell death. In this case, radionuclides are conjugated via a linker to a monoclonal antibody in order to deliver a dose of radiation specifically to tumor cells expressing a specific cell-surface antigen [reviewed in (161)]. Although a promising area of tumor therapy, RIC therapy has several limitations, due to the radioactive conjugates involved. The pharmacokinetic biodistribution is dependent on the conjugated antibody and this leads to a dose rate two orders of magnitude below conventional external beam radiation therapy, which may limit the use of RICs (162). Although this type of therapy has shown promise in several cancer types, there are no published studies in lung cancer thus far.

## Conclusions

Lung cancer is a highly unstable cancer with genomic instability being a primary driver of the disease. The highly genetically diverse lung cancers are driven by the exposure to DNA damage from both exogenous and endogenous sources. Although the loss of the normal DNA repair machinery and accumulation of mutations initially drives the tumor progression, it also provides a targetable defect for therapeutic intervention. Such agents that target these defects have been shown to be effective against lung cancer, including combining traditional therapies, such as platinum agents and radiotherapy, and more recently with targeted therapies, such as immunotherapies. Future research efforts are likely to involve refining these combination treatments to overcome the development of tumor resistance to treatments and to improve survival outcomes for patients. Further study of agents that target DNA damage and repair pathways, such as PARP1 inhibitors and ADCs linked to DNA damaging agents are vital to determine their regulatory and subsequent approval for use in the clinic. It is also likely that the further characterization of DNA repair proteins and pathways will drive the quest for new lung cancer therapeutics targets in subsequent years.

## Author Contributions

JB, MR, DB, JP, CM, MF, CO'L, DR, KO'B, and EB contributed to the writing and editing of the manuscript. MR made the figures. All authors contributed to the article and approved the submitted version.

## Conflict of Interest

KO'B and DR are founders of CARP Pharmaceuticals. EB, DR, and KO'B are founders of Carpe Vitae Pharmaceuticals. EB, JB, KO'B, and DR are inventors on patent applications filed by Queensland University of Technology. The remaining authors declare that the research was conducted in the absence of any commercial or financial relationships that could be construed as a potential conflict of interest.
